# Control of Germinal Center Responses by T-Follicular Regulatory Cells

**DOI:** 10.3389/fimmu.2018.01910

**Published:** 2018-08-24

**Authors:** James B. Wing, Murat Tekgüç, Shimon Sakaguchi

**Affiliations:** ^1^Laboratory of Experimental Immunology, Immunology Frontier Research Center, Osaka University, Suita, Japan; ^2^Department of Experimental Pathology, Institute for Frontier Medical Sciences, Kyoto University, Kyoto, Japan

**Keywords:** regulatory T-cells (Tregs), T follicular helper (Tfh) cell, T follicular regulatory (Tfr) cell, germinal center (GC), autoimmunity

## Abstract

Regulatory T-cells (Treg cells), expressing the transcription factor Foxp3, have an essential role in the control of immune homeostasis. In order to control diverse types of immune responses Treg cells must themselves show functional heterogeneity to control different types of immune responses. Recent advances have made it clear that Treg cells are able to mirror the homing capabilities of known T-helper subtypes such as Th1, Th2, Th17, and T-follicular helper cells (Tfh), allowing them to travel to the sites of inflammation and deliver suppression *in situ*. One of the more recent discoveries in this category is the description of T-follicular regulatory (Tfr) cells, a specialized subset of Treg cells that control Tfh and resulting antibody responses. In this review we will discuss recent advances in our understanding of Tfr biology and the role of both Tfr and activated extra-follicular Tregs (eTreg) in the control of humoral immunity.

## The humoral immune response

Antibody driven humoral immunity is essential for host protection from a range of pathogens. This can be broadly separated into the T-independent response, in which B-cell subsets such as B1 cells and marginal zone B cells produce low-affinity antibodies that allow a rapid response to infection, and the T-dependent response in which T-cell help allows the generation of high-affinity antibody and memory immunity over a longer period. Of key importance to T-dependent antibody responses is the germinal center, a structure formed by follicular B-cells and dependent on T-cell help. The germinal center itself is segregated into a dark zone, where centroblast B-cells undergo rapid proliferation and somatic hypermutation (SHM), and the light zone, where higher affinity B-cells are selectively helped by T-cells, allowing them to survive and either be selected as memory or plasma cells, or be recycled back to the dark zone for further rounds of SMH (1). CD4^+^ T-follicular helper (Tfh) cells play a critical role in this process as they are responsible for the majority of T-cell help given to follicular and germinal center B-cells, via delivery of CD40 and IL-21 stimulation to B-cells (2). Tfh form through a multistage differentiation process initiated by contact between dendritic cells (DCs) and pre-Tfh CD4^+^T-cells. This alone is insufficient to stabilize the full Tfh program, and a second step of prolonged contact between antigen-specific B-cells and the pre-Tfh cell is then required to allow progression to the mature Tfh phenotype. Following this, the Tfh cell can then further differentiate into a highly-activated and germinal center-resident GC-Tfh cell distinguished by high-level expression of CXCR5 and PD-1, in contrast to intermediate levels of both markers expressed by Tfh (2). The chemokine receptor CXCR5 allows trafficking of the Tfh cell into the B-cell follicle as its ligand CXCL13 is produced by follicular resident dendritic cells and, in humans, by Tfh themselves, allowing further recruitment of new Tfh. Due to their critical role in the generation of high-affinity antibody responses, Tfh cells are vital for the generation of effective humoral immunity. However, dysregulation and unchecked activation of Tfh cells or germinal centers in both humans and mice lead to the production of autoantibodies and lupus-like symptoms, demonstrating the need to tightly regulate the function of these cells (3–5). Additionally, due to their highly mutational nature, germinal centers themselves are a common source of tumorigenesis, meaning that even foreign antigen-reactive germinal center cells require tight regulation (6).

## Tregs and Tfr cells

Regulatory T-cells (Tregs) expressing the transcription factor Foxp3 are critical for the maintenance of immune homeostasis (7). Signs of a link between certain T-cell populations and the control of humoral immunity have been present since the foundational work that first hinted at the presence of a T-cell population that regulated immunity, demonstrated through the inhibition of anti-sheep red blood cell antibody responses by thymically-derived populations (8). Later, in work leading up to the formal discovery of Tregs, we found that autoantibodies were one of the most sensitive indicators of T-cell autoimmunity (9). When we identified Tregs on the basis of their CD25 expression we found that anti-CD25 depletion of Tregs lead to strong induction of autoantibodies against parietal cells in the stomach epithelia, and against thyroglobulin proteins produced by thyroid follicular cells (10). While CD25 is not entirely exclusive to Tregs, specific depletion of Tregs via diphtheria toxin in mouse models in which Tregs express the primate diphtheria toxin receptor leads to strongly-enhanced GC formation, Tfh cell expansion and antibody responses (11, 12). Loss of control over humoral immunity is also characteristic of mutations of Foxp3 in the scurfy mouse strain and in immune dysregulation, polyendocrinopathy, enteropathy, X-linked (IPEX) syndrome patients, and leads to the production of autoantibodies, hyper IgE and strongly-enhanced GC/Tfh responses (12–18)

Tregs themselves comprise a number of subpopulations, with some functionally-specialized groups mirroring the transcriptional programming of effector T-cell subsets, allowing them to gain expression of the chemokine receptors responsible for localization to the sites of inflammation in order to suppress the mirrored effector population (19). Early work suggested that, following activation, CD4^+^CD25^+^CD69^−^ Tregs are capable of gaining CXCR5 expression while losing CCR7, a chemokine receptor that homes to the T-cell zone, allowing them to travel to the B-cell follicle to suppress B-cell responses (20, 21). However it was not until 2011 that three groups described Tfr in detail and defined them as CXCR5^+^PD-1^+^BCL6^+^Foxp3^+^ cells (15, 22, 23). While Tfr differentiation is not as well characterized as Tfh, evidence thus far suggests that they have a similar developmental path, with both undergoing a multistage differentiation process dependent on signals such as CD28, ICOS, SAP and B-cell contact and the Tfh transcription factor BCL6 (15, 22).

Tfr present in the lymphoid organs are an induced subset of effector Tregs. As a result, in a healthy mouse kept in pathogen-free conditions, Tfr are present only in very small numbers in the spleen and lymph nodes, although they can be found in significant numbers in sites of ongoing humoral immune responses, such as the Peyer's patches. In significant contrast to Tregs, Tfr downregulate the IL-2 receptor alpha chain, CD25 (24–26). Downregulation of CD25 appears to be a marker of Tfr development, with CD25^+^ Tfr forming initially, before later formation of more highly-differentiated CD25^−^ Tfr. Microscopic analysis of Tfr in the spleens and draining lymph nodes of vaccinated mice reveals that, while the majority of Tfr resident in the follicle and near the T-B border express CD25, almost all germinal center-resident Tfr lack CD25 expression (26). Accordingly, detailed analysis of chemokine receptor and cell adhesion molecules demonstrates that, in keeping with their germinal center localization, CD25^−^ Tfr express significantly increased levels of CXCR5, while reducing expression of molecules, such as CCR7 and PSGL-1, responsible for maintenance of localization in the T-cell zones. Further detailed characterization by flow cytometry and RNA-sequencing shows that while CD25^+^ Tfr are more similar to effector Tregs, CD25^−^ Tfr have shifted their gene expression signature to a point equidistant between Tfh and effector Tregs, displaying a high level of flexibility in their phenotype. Despite this, they retain stable expression of Foxp3, maintain a characteristic Treg epigenetic signature, and express key Treg suppressive molecules such as CTLA-4, allowing them to suppress both T-cells and B-cells during *in vitro* co-culture (26). Due to their relative similarity to Tfh, it is reasonable to ask if Tfr are formed from thymically-derived Tregs or peripheral Tregs, potentially due to Tfh conversion into Tfr. However, following adoptive cell transfer, both CD25^+^ Tfr and CD25^−^ Tfr are formed from naïve CD25^+^Foxp3^+^Tregs, and in agreement with earlier studies showed little evidence that they can form from transferred CD25^−^ Foxp3^−^ T-cells (15, 22–24, 26).

**Figure 1:**
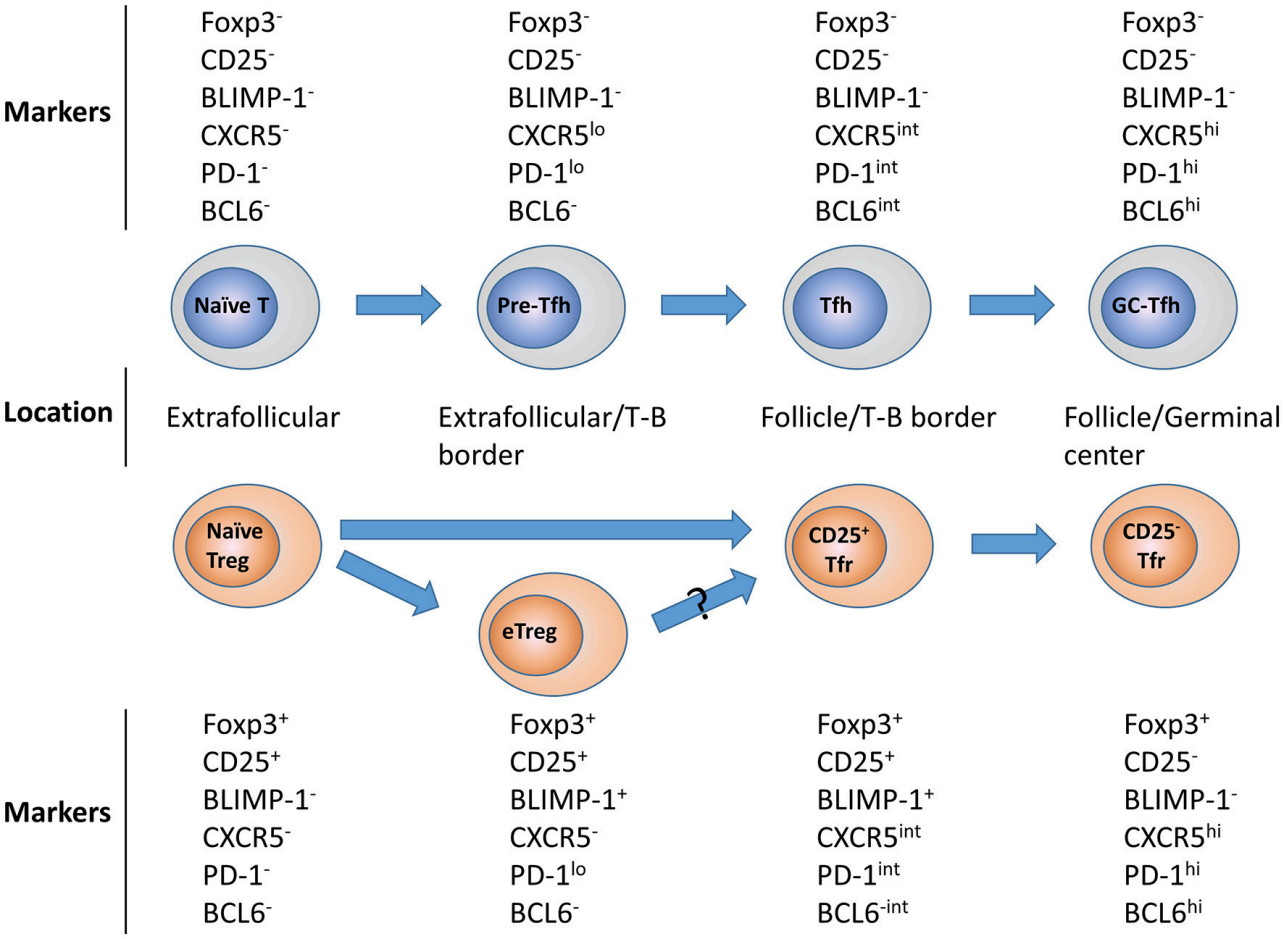
Tfr and Tfh differentiation. Upon activation naïve CD25^+^ Tregs differentiate into activated effector Tregs in the T-cell zone or non-lymphoid tissues or early follicular resident CD25^+^Tfr. These CD25^+^Tfr can them downregulate CD25 expression causing the loss of BLIMP-1 expression and higher level BCL6 and CXCR5 expression, allowing these CD25^−^ Tfr to travel to the germinal center itself. All cell depicted are CD3^+^CD4^+^. Corresponding development of Tfh is also shown for contrast.

While CD25^+^ Tfr in the mouse appear to be at an earlier stage in their differentiation, they are still identifiably Tfr due to their expression of a range of markers at intermediate levels such as CXCR5, PD-1, and BCL6, and localization in the B-cell follicle. As a result of this, we propose a model, in which following initial stimulation, a naïve Tregs bifurcate into eTregs or CD25^+^ Tfr in the follicle, before receiving further activation which allows them to become terminally-differentiated germinal center-resident CD25^−^Tfr. This suggests that in the mouse, CD25^+^ Tfr and CD25^−^ Tfr may be the Treg equivalents of Tfh and GC-Tfh, respectively (Figure 1).

A critical question raised by these findings is—why do terminally differentiated Tfr lose CD25 expression? CD25 was the molecule by which Tregs cells were first clearly identified, and is considered both a canonical marker and a critical component for normal Treg function (27). In contrast, IL-2 is known to inhibit Tfh responses, due to STAT5-induced upregulation of BLIMP-1, which inhibits expression of the critical Tfh transcription factor BCL6 (28–30). A further factor to consider is that BLIMP-1 is expressed by many effector Tregs and plays an important role in their suppressive function by regulating expression of a range of genes such as IL-10 (31, 32). Since Tfr are also a form of effector Treg, this suggests they must maintain a fine balance of these potentially conflicting factors to maintain their phenotype. We and several other groups have demonstrated that addition of IL-2 alongside vaccination or infection in mice inhibits the formation of CD25^−^ Tfr cells while at the same time causing expansion of Tregs (24–26). This is due to a BLIMP-1-dependent mechanism, in which IL-2 causes increased expression of BLIMP-1, which represses expression of BCL6, thus inhibiting Tfr formation (24). As a result CD25^−^ Tfr express only low levels of BLIMP-1 but high BCL6, while CD25^+^Tfr express higher BLIMP-1 but have only intermediate levels of BCL6 (24, 26). This changing role for IL-2 marks a fundamental split in Treg identity, with the majority of tissue-resident effector Tregs having a BLIMP-1- and IL-2-dependent identity, while fully-differentiated CD25^−^ Tfr depend on BCL6 and are thus inhibited by IL-2. CD25^−^ Tfr can instead be maintained by the presence of other cytokines and signals such as IL-4, which is highly produced by Tfh (2, 26). It is also the case that CD25^−^CXCR5^−^BCL6^−^Foxp3^+^ Tregs at tissue sites of inflammation can be maintained in an IL-2 independent manner (33).

While it is clear that a large proportion of Tfr downregulate CD25 in mice, recent results examining human Tfr suggest that downregulation of CD25 may be less characteristic of human Tfr. Sayin et al. demonstrate via microscopy that the majority of Tfr detectable in the follicles of human mesenteric lymph nodes express CD25, and that the cells are highly concentrated at the T-B border but not the GC itself (34). Interestingly, while microscopy suggested that essentially all the Tfr in the B-cell follicle and GC itself were CD25^+^, flow cytometry analysis in the same report demonstrates that PD-1^hi^ Tfr express significantly less CD25 than PD-1^int^ or negative Tfr (CD25 MFI 616 ± 96 vs. 1101 ± 121.4, *p* = 0.0074 unpaired *t*-test), and also display a bimodal expression of CD25 with a significant fraction appearing to be CD25^lo/−^ (34). This is in keeping with two previous reports that suggested that the most highly-differentiated PD-1^hi^CXCR5^hi^BCL6^+^ Tfr in human tonsils also downregulate CD25 (25, 26). Importantly, however, while PD-1^hi^ Tfr do appear to be enriched in the GC itself, they are extremely rare, with only around 3% of Tregs in the mLN matching this description (34). Similarly we found around 5% in the tonsils (26). As a result the ratio of Tfh/Tfr is skewed heavily to Tfh in the GC region, which may indicate that Tfr outside the GC itself are most critical in humans (34) and may also explain why human lymph node resident-Tfr are resistant to rituximab induced depletion of GC B-cells (35). In contrast, the mouse appears to have a greater number of Tfr in the GC itself, and a correspondingly larger fraction of CD25^−^ Tfr.

IL-21, a characteristic Tfh cytokine, may play a role in the maintenance and differentiation of Tfr. IL-21 has been demonstrated to indirectly affect Tregs homeostasis by suppressing IL-2 production by Tconv cells (36). However, more recently, cell-intrinsic roles for IL-21 on the formation of Tfr have been described (37, 38). Autoimmune-prone BXD2 mice lacking IL-21 production have their Tfh/Tfr ratio skewed toward Tfh. This appears to be due to both direct effects on Tfh STAT3 signaling, and possibly indirectly via Akt signaling in Tfr (37). Jandl and colleagues found that Tregs lacking IL-21R have an increased proportion of Tfr among total Tregs. Further, the proportion of Tfr that express CD25 was increased by a reduction in IL-21, which would otherwise induce BCL6-mediated downregulation of CD25 expression. When IL-21R-deficient or WT Tregs were transferred into Treg-depleted mice, followed by vaccination, loss of IL-21R expression by Tregs was associated with reduced antigen-specific antibody production. Interestingly, this loss of antigen-specific antibody was marked by a reduction in the percentage of antigen-specific B-cells within the GC but no change in the total number of GC-B-cells. While it was not examined in this case, this would imply that there was a proportional gain in non-antigen-specific or autoreactive B-cells in the same system (38). As a result it seems that IL-21 can prevent BCL6-driven downregulation of CD25, and thus enhance IL-2-driven Tfr proliferation. However, as noted earlier, IL-2 itself inhibits Tfr differentiation via BLIMP-1-dependent inhibition of BCL6 (24–26). These results may seem contradictory, however a key point here is to indicate the split between CD25^+^ and CD25^−^ Tfr. We found that supplementation with IL-2 results in an almost total loss of CD25^−^ Tfr but CD25^+^ Tfr are retained. Equally, CD25^+^ Tfr are preferentially expanded in IL-21R-deficient Tregs. This suggests that IL-21 and IL-2 may control the balance between CD25^+^ and CD25^−^ Tfr, since IL-2 enhances the proliferation of naïve and eTregs (which are the precursors of Tfr) and CD25^+^ Tfr in the follicle, while blocking full differentiation into germinal center-resident CD25^−^ Tfr. On the other hand, IL-21 may encourage CD25^+^ Tfr to fully differentiate into CD25^−^ Tfr via its effects on BCL6-mediated downregulation of CD25, but this comes at the price of reduced IL-2-dependent proliferation by CD25^+^Tfr.

## The *in vivo* role of Tfr and contribution of tregs to humoral immunity

Studies into the exact *in vivo* role of Tfr have yielded conflicting results. Several initial studies used adoptive transfer systems to study the function of Tfr. Here, they transferred CXCR5- or BCL6-deficient Tregs into T-cell-deficient mice, alongside WT CD4^+^Foxp3^−^ cells, before vaccinating them. Loss of Tfr function in this system caused an increase in the number of germinal center B-cells while also increasing the amount of antigen-specific antibody, albeit with reduced affinity (15, 23). Another study used bone marrow chimeras of SAP-deficient and Foxp3-deficient bone marrow. These mice lack Tfr, since the Foxp3-sufficient cells lack SAP, which is critical for Tfr development. In this system, GC and Tfh numbers were increased but antigen-specific antibody production was reduced, presumably due to increased expansion of self-reactive Tfh (22). Treg-specific inhibition of TRAF3-dependent ICOS expression and a resulting defect in Tfr formation caused an increase in the number of GC B-cells, with no change in Tfh cell number but rather increased cytokine production by these cells which in turn also resulted in increased SHM (39). Reduced Tfr infiltration into the follicles due to loss of NFAT2-dependent CXCR5 expression also resulted in increased GC cell numbers and antigen-specific antibody production (40). The transcription factor Helios is also expressed by the majority of Tregs and Tfr. Mice with a Treg-specific loss of Helios expression develop autoimmunity characterized primarily by enhanced autoantibodies, GC size and Tfh cell number. This appears to be primarily due to loss of Tfr cell function, although some other abnormalities, such as an unstable phenotype and gain of pro-inflammatory cytokine production by Tregs, suggests that this phenotype may also be partly attributable to a wider loss of Treg function (41).Further to this, recent work demonstrates an essential role for mTOR complex 1 (mTORC1) signaling that induces STAT3-TCF1-driven induction of BCL6 expression (42). As a result, when essential components of the mTORC1 pathway were genetically depleted, Tfr development and function were impaired, leading to enhanced numbers of GC B-cells and Tfh following vaccination.

Altogether, these results suggest that Tfr control GC cell number and Tfh function but have varied effects on the quality and antigen specificity of the response. Conditional knockout of BCL6 in Tregs via the *cre-lox* (BCL6-flox: Foxp3-cre) system promises the ability to analyse Tfr function in more detail than is possible with cell transfer or bone marrow chimera approaches, and without some of the caveats that come with loss of function in genes that may also affect broader Treg functionality. Using this system, the Dent group found that Tfr were, as expected, significantly reduced, but this had no effect on either GC B-cell or Tfh cell numbers. However, Tfh production of IFN-γ, IL-10, and IL-21 were increased, resulting in increased IgA production but a reduction of IgG in the context of vaccination with sheep red blood cells or NP-KLH. In contrast, in pristine induced lupus models, dsDNA autoantibody specific IgA was increased in the absence of a clear effect on IgG. However, when using the same mouse model with a DNA prime-protein boost vaccination, the IgG titer was not affected but antibody avidity was reduced, suggesting a role for Tfr in the control of the quality of the antibody reaction (43). In contrast, another group demonstrated that Tfr are critical to IgA selection in the gut and that their presence increases the production of IgA-positive plasma cells in the lamina propria. This in turn has a critical role in the regulation of the microbiota via IgA (44). Further work in an influenza virus infection system demonstrates that, in this situation, Treg-specific BCL6 deficiency induced no change in GC and Tfh cell numbers, but caused a clear increase in the number of antibody-secreting plasma cells. However, this also coincided with a reduction in the proportion of antigen-specific cells, while increased autoantibody production was also observed when the infected mice were treated with IL-2 in order to suppress Tfr function (24). These findings were recently built on, with another group using BCL6-flox Foxp3-cre mice to demonstrate that—again—loss of Tfr had no clear effect on GC or Tfh cell number in influenza infection, but that influenza specific IgG2c antibody production was slightly, but significantly, increased (45). While these mice were healthy at a young age, by 30 weeks they had developed immune infiltration of several organs such as the lung, pancreas, and salivary gland, while also developing autoantibodies. In contrast to influenza infection, increased numbers of GC and Tfh were seen, suggesting that Tfr may control self-reactive GCs to a larger extent than non-self-reactive responses (45).

While it is clear that Tfr play an important role in the control of antibody production, whether they primarily control GC B-cell numbers or have a subtler role in the control of the quality and specificity of the antibody response remains less clear. Recent studies using conditional genetic deletion of BCL6 in Tregs do not suggest a role for Tfr in the control of overall GC numbers during the response to foreign antigens, but instead see a more subtle role in modulating antibody production via plasma cells. In contrast, in the context of a primarily self-antigen-driven response, Tfr may play a more active role directly controlling GC B-cell and Tfh cell numbers. This may be because Tfr have a more self-skewed TCR repertoire, respond better to vaccination with self-antigens rather than foreign antigens, and do not appear to require recognition of the same antigen as a particular Tfh cell in order to suppress it (25, 46, 47). We also previously demonstrated that while short-term depletion of Tregs/Tfr is effective at enhancing antigen-specific Tfh formation following vaccination with a foreign antigen, longer-term depletion of Tregs further increased the total number of Tfh but also reduced the absolute number of antigen-specific Tfh (12). This indicates that while partial or temporary disruption of Treg/Tfr function leads to increased availability of co-stimulatory molecules, and a resulting increase in the antigen-specific immune response, prolonged or total disruption of Treg/Tfr function may lead to a more profound loss of immune homeostasis resulting in aggressive expansion of autoreactive cells, which outcompete antigen-specific cells and cause skewing to self-reactive responses. As a result it is possible that a more or less complete loss of function in Tfr may have differing effects.

**Figure 2:**
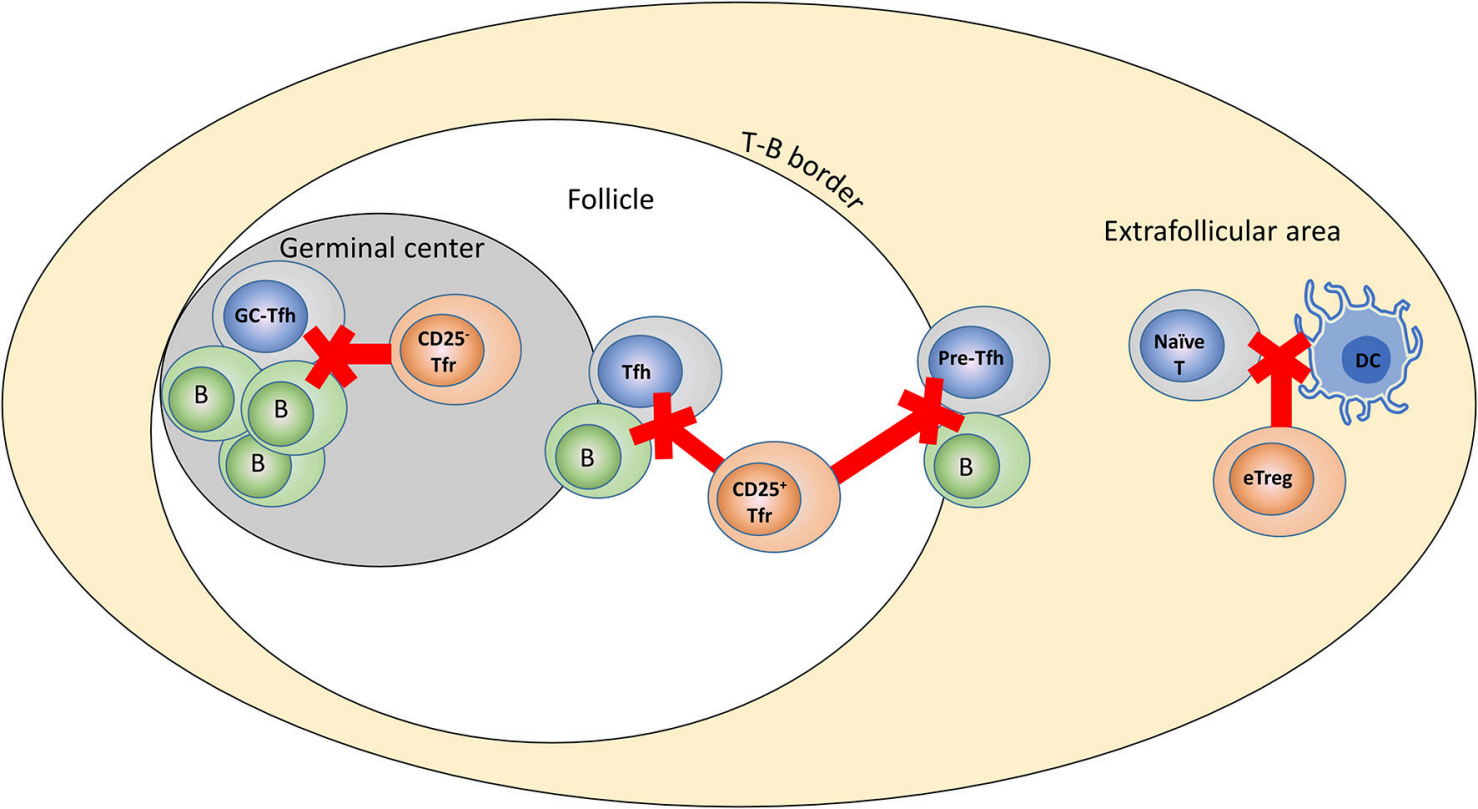
Role of different Treg subsets in control of the GC response. Model of potential differing roles for Tfr and Tregs in the control of humoral immunity. Tregs control the initial interaction of naïve T-cells with DCs, CD25^+^ Tfr control interactions at the T-B border and travel through the follicle, while CD25^−^ Tfr are responsible for direct suppression in the GC itself.

The relatively subtle effects of specific Tfr depletion stand in considerable contrast to the large effects seen when Tregs as a whole are depleted (11, 12). Equally, while the most highly-differentiated Tfr lack CD25 expression, anti-CD25 antibody is capable of inducing substantial autoantibody production (10). Given that it takes several days for Tfr to form following initial stimulation it is reasonable to surmise that Tregs outside the follicle are responsible for the control of the initiation stages of Tfh formation, while Tfr may be more critical a later in the process. We suggest a model in which CXCR5^−^ Tregs, CD25^+^ Tfr present in the follicle and T-B border, and CD25^−^ Tfr present in the GC have distinct roles at different points following initial stimulation of a GC reaction, essentially forming three rings of protection for the prevention of autoreactive GCs (Figure 2). Specifically, CXCR5^−^ Tregs may control the initial formation of Tfh via suppression of the contact between DCs and n naïve T-cells, CD25^+^Tfr in the follicle may interfere with contact and signaling between Tfh and B-cells at the T-B border or during transit through the follicle, while CD25^−^ Tfr resident in the GC itself may interfere with the interactions between GC-Tfh and centrocytes. This division is likely to be temporal as well as spatial, with the key events in the extra-follicular region occurring earlier than events in the GC. Even in the context of an established GC reaction, the outer rings of defense may still be critical to prevent new autoreactive cells infiltrating an existing GC as given sufficient antigen naïve B-cells and newly formed Tfh are capable of entering pre-existing GCs even at relatively late stages of their life cycle (48, 49). The lines between these rings of defense are likely to be blurred for several reasons: Tregs, CD25^+^ Tfr and CD25^−^ Tfr are developmentally related and a single cell could potentially perform in all three areas over the course of its differentiation, also, almost all Tfr in the GC lack CD25 expression the follicle contains a mixture of CD25^+^ and CD25^−^Tfr which may represent GC Tfr cells traveling back to the follicle in a manner similar to Tfh (48). With these caveats in mind, we believe this model may capture the essence of the division of labor between these Treg subsets. While loss of CD25 appears to be a good marker of Tfr differentiation in mice, in humans CD25^−^ Tfr located in the GC seem to be rare, so it may be the case that human Tfr are weighted to a greater role for CD25^+^ Tfr in the B-cell follicle and T-B border (34). As a result this model may be a good fit for the murine system while further detailed experiments are required to better understand human Tfr biology.

## Mechanisms of Tfr function

A number of suppressive mechanisms have been proposed to have a role in Tfr function. CTLA-4 is known to be critical to Treg suppressive function to the extent that its specific deletion in Tregs leads to severe autoimmunity similar to that seen in Foxp3-deficient scurfy mice (50). We and others previously demonstrated that loss of CTLA-4 function in Tregs had a severe impact on the suppression of Tfh responses (12, 51, 52). CTLA-4 primarily acts to deplete the CD28 ligands CD80 and CD86 from the surface of antigen-presenting cells, preventing them from providing co-stimulation to T-cells (53). It is likely that this mechanism is of primary significance in controlling the initial stages of Tfh cell formation at the T-B border, since blockade of CD80 and CD86 has little effect on already preformed Tfh cells, while mice lacking CD80 and CD86 on B-cells but not DCs were still able to form Tfh and GCs, suggesting that the core function cell extrinsic function of CTLA-4 may be at the early stage of GC formation during contact with DCs (54, 55). However, several groups have also found that expression of CD80 and CD86 by B-cells alone are sufficient to induce germinal center reactions in mice otherwise lacking CD80 and CD86 (12, 56). In both cases, a cell-intrinsic role for B-cell CD80 and/or CD86 was described, since when transferred together CD80/86 sufficient B-cells were better able to forms GC B-cells than CD80/86 deficient B-cells (12, 56). Further to this, a B-cell-intrinsic role for CD80 in controlling Tfh and plasma cell formation was also observed (57). Together these results would suggest that in specific circumstances, CD80 and CD86 expression on either DCs or B-cells may be dispensable but that it is likely that optimal Tfh responses require both. Aside from its ligand-depleting function, CTLA-4 has also been suggested to mediate direct suppression of B-cell antibody production by putative Tfr (CD25^+^CD69^−^), although the molecular events underpinning this remain unclear (21). It is also possible that cell-intrinsic functions of CTLA-4 that act on the Tfr itself may have a role in the control of later-stage GC reactions (51) as at least some of the effect of CTLA-4 appears to be independent of CD28 signaling (58).

Another recently proposed mechanism of Tfr function is expression of the IL-1 decoy receptor IL-1R2 (25). Addition of IL-1 to vaccinated mice enhances the Tfh response and resulting antibody production, while blocking IL-1 has the opposite effect. Tfr have enhanced IL-1R2 expression in comparison to other Tregs and are able to inhibit IL-1 driven enhancement of Tfh cytokine production in a similar manner to blocking IL-1 (25). This suggests that Tfr may be able to engage IL-1 and prevent its interaction with Tfh cells. RNA sequencing reveals that IL-1R2 is expressed by both CD25^+^ and CD25^−^ Tfr (26). Further work is required to determine the *in vivo* importance of this proposed mechanism. Additionally, IL-1R2 is also highly expressed by both tumor-infiltrating and CXCR3^+^T-bet^+^ pancreatic Tregs, suggesting that it may be a mechanism used by a range of highly-activated Tregs subtypes, including Tfr (59, 60).

GARP, a Treg surface molecule that supports the anchoring of latent TGFβ onto the surface of Tregs, has also recently been shown to be enriched on the surface of human Tfr, although again further work is required to determine its exact *in vivo* contribution to Tfr function (34). TGFβ signaling has been shown to control Tfh numbers, although the phenotype is much more severe than loss of Tfr function alone, suggesting that it may be involved at multiple stages of Tfr/Treg function (61).

Tregs have also been suggested to directly kill activated B-cells via production of granzymes and perforin (62, 63). This work was carried out before the full description of Tfr, but it seems likely that these cells were primarily naïve/eTregs since they were purified on the basis of CD25 expression from healthy wild-type mice.

Surprisingly, recent work suggests that Tfr may also have some positive role in the modulation of the GC response. Some earlier results suggest that Tfr may support specific IgG production and affinity maturation in at least some contexts (22, 43). Building on this, recent results demonstrate that production of the cytokine IL-10 by Tfr enhances germinal center responses by driving germinal center B-cells into a proliferative dark zone phenotype via induction of the transcription factor FOXO1. As a result, specific knockout of IL-10 in Tfr results in reduced GC cell numbers (64). In contrast, IL-10 receptor-deficient T-cells more readily develop into Tfh cells, suggesting that IL-10 signaling into B and T-cells may have differing effects on GC and Tfh formation (65). The finding that Tfr have at least some ability to support the germinal center reaction differs from the results from models that look at total loss or inhibition of Tfr function, and suggests that while their overall contribution can probably be characterized as suppressive, Tfr may have a complex role in the fine-tuning of the germinal center response that includes the delivery of some conflicting signals (66).

**Figure 3:**
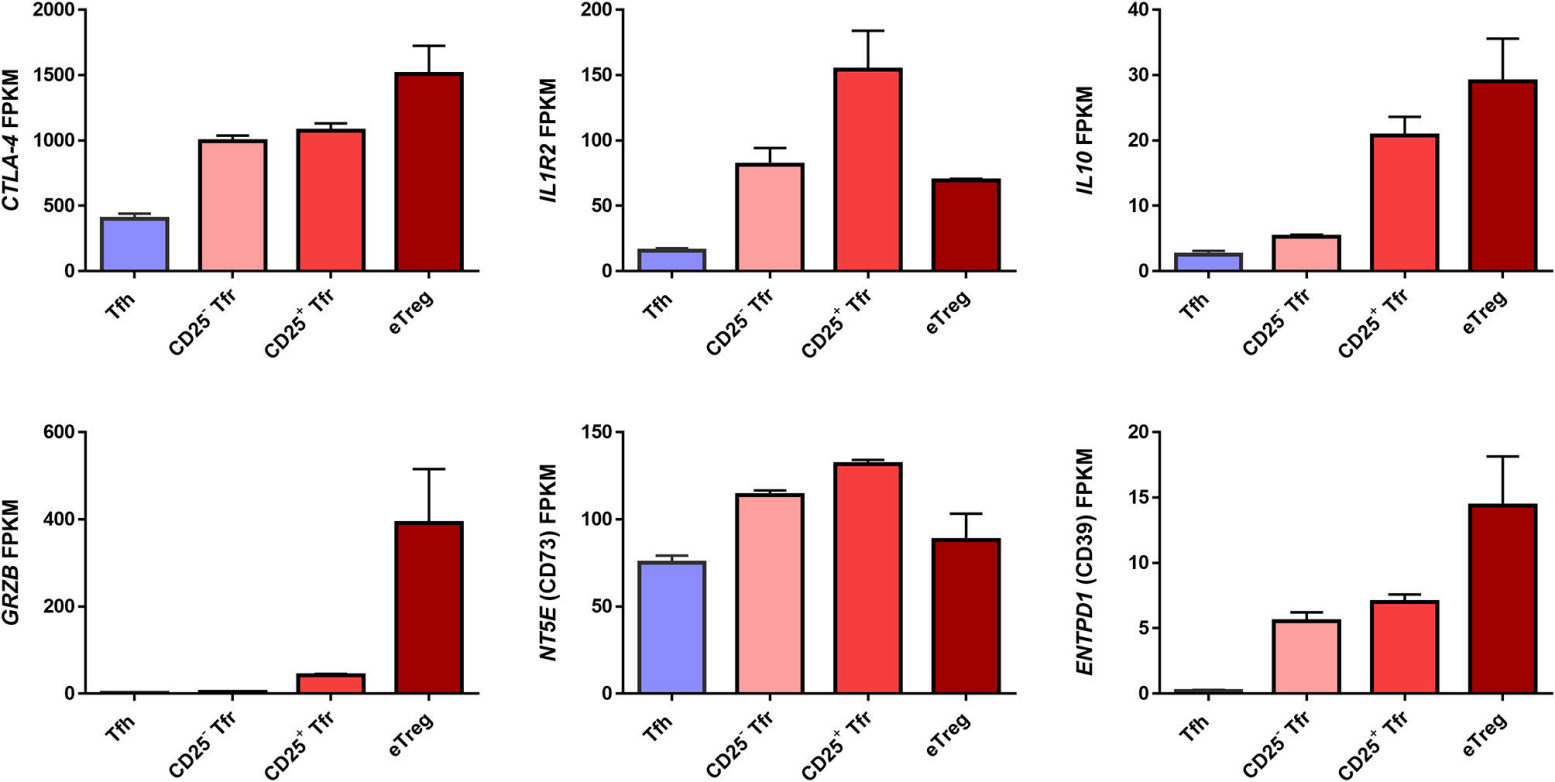
Expression of suppressive genes by Tfh, Tregs, CD25^+^ Tfr, and CD25^−^ Tfr. RNA expression of murine Tfr cells measured as Fragments Per Kilobase Million (FPKM) taken from RNAseq dataset in Wing et al. (26). *n* = 2 ±SEM. Mice were vaccinated with NP-Ova in alum and cells sorted from peripheral lymph nodes 7 days later. CD4+B220– cells from Foxp3 reporter were sorted as CD62L^−^CXCR5^+^PD1^+^Foxp3^−^GITR^−^ Tfh, CD62L^−^CXCR5^−^Foxp3^+^GITR^+^CD25^+^ eTreg, CD62L^−^CXCR5^+^PD1^+^GITR^+^CD25^+^ CD25^+^ Tfr, and CD62L^−^CXCR5^+^PD1^+^Foxp3^+^GITR^+^CD25^−^ CD25^−^ Tfr.

In short, a number of potential mechanisms of Treg/Tfr control of humoral immunity have been identified. Further analysis of RNA sequencing data obtained from Tregs, CD25^+^ Tfr and CD25^−^ Tfr revealed that these cells have differing expression of various Treg suppressive molecules (26) (Figure 3). Importantly we use activated (CD44^+^CD62L^−^CXCR5^−^) but not Naïve Tregs as a baseline comparison to Tfr. Since Tfr themselves are all CD44^+^CD62L^−^ their direct comparison to total CXCR5- Tregs which contain a significant proportion of naïve cells will lead to the identification of a range of effector Treg markers being misidentified as Tfr enriched. Granzyme B appears exclusive to eTregs, IL-10 is present in CD25^+^ Tfr and eTregs, IL1-R2 is concentrated in the CD25^+^ Tfr, CD73 is slightly increased in both Tfr subsets, CD39 and CTLA-4 appear to favor eTregs. However only IL-10 and granzyme B were identified as differentially expressed between these Treg groups, while CTLA-4 protein was also confirmed to not be significantly different by flow cytometry (26). In all cases Tfh themselves express lower levels of these molecules. As a result, it seems Tfr may act via a number of suppressive mechanisms and that all of these functional molecules are shared with Tregs.

While the mechanisms of Tfr function are still under investigation, it is of interest that Tfr induce sustained suppression of Tfh and B-cells that outlasts the immediate contact between these cells. B-cells that have been previously suppressed by Tfr have altered metabolic and epigenetic programming that impair their ability to respond to later stimulation by Tfh cells in the absence of Tfr cells (67). This suggests that the effect of Tfr suppression persists beyond the actual contact period between the Tfr and B-cell/Tfh, and may be critical in the generation of a large effect from a small number of cells, since Tfr are significantly outnumbered by B-cells and Tfh in the follicular environment, particularly in human GCs (34).

## Circulating Tfr

While bona-fide Tfr are defined by their localization in the B-cell zones of lymphoid organs, CXCR5^+^Foxp3^+^ circulating Tfr (cTfr) can also be found in blood. Due to the relative difficulty in obtaining samples of human lymphoid tissues, this population has been the focus of investigations in humans.

cTfr in mice appear to be formed early in the Tfr differentiation process since they are retained even in μMT B-cell deficient mice, suggesting that they are dependent on the initial contact with DCs but do not require the second contact with B-cells that would normally finalize the differentiation process (46, 68). Similarly in humans cTfr cells were generated in the peripheral lymphoid tissue following the initial activation mediated by DCs, but were similarly not affected by a lack of B-cells demonstrating an early bifurcation of cTfr and tissue-resident Tfr (68). As a result there are significant phenotypic differences between cTfr and from Tfr isolated from tonsils and other lymphoid organs with cTfr having much reduced expression of normally characteristic markers such as PD-1, ICOS and BCL6 (26, 46, 68, 69). This difference is further emphasized by the finding that Tfr from human tonsils have a subset of CD25^negative^/^lo^ BCl6^+^Foxp3^+^ Tfr while CD45RA^−^ effector Tfr in blood are CD25^int^. In contrast CXCR5^−^ effector Tregs in blood upregulate CD25 in comparison to naïve Tregs, again demonstrating the significant difference in IL-2 metabolism between Tfr and other effector Tregs (26). Interestingly due to their CD25^int^ nature CD45RA^−^ cTfr find themselves in the fraction (FRIII) of blood Tregs that we previously identified as primarily Foxp3^int^CD25^int^ non-Tregs (70). However, since we and others have confirmed that cTfr retain stable expression of Foxp3, a Treg-type demethylation signature, and suppressive function, it seems that FRIII may contain a mixture of Tregs and non-Tregs (26, 68, 71). This mixed nature of FRIII was recently confirmed by the finding that CD127^−^CD25^int^CD45RA^−^ FRIII Tregs can be further divided into CD49d^+^CCR4^−^, CCR4^−^CD49d^−^, and CCR4^+^CD49d^−^ cells, with the CD49d fraction expressing inflammatory cytokines (72). In our hands CD45RA^−^CD127^−^CXCR5^+^CD25^Int^ cTfr lack CCR4 and CD49d expression and, as a result, cTfr make up the majority of CCR4^−^CD49d^−^ cells in FRIII (26). This suggests that FRIII can be stratified into CD49d^+^CXCR5^−^CCR4^−^ non-Tregs, CD49d^−^CXCR5^+^CCR4^−^ cTfr and CD49d^−^CXCR5^−^CCR4^+^ Tregs.

Surprisingly, given that CXCR5 is normally considered an activation/memory marker in T-cells, some Tfr found in peripheral blood appear to have a naïve phenotype (CD45RA^+^CXCR5^+^) (68, 69, 73). Similar to Tfr themselves, the first indication of the presence of these cells can be found in the work of Lim et al. in 2006 who found that, in contrast to a range of other chemokine receptors normally associated with effector/memory cells, such as CCR2, CCR4, and CCR6, CXCR5 expression by Tregs was increased on CD45RA^+^ cells in a similar manner to CCR7 (73). Expression of markers such as CD45RA would normally be considered an indication of naïve status. However, these cells are absent in cord blood and the thymus, suggesting that they are induced from truly naïve Tregs by stimuli that occur after birth (68, 73). Given that CD45RA^+^ Tregs are now considered a promising target for *in vitro* expansion and clinical use, this phenomenon may require further investigation (74).

While it is clear that cTfr retain the ability to suppress T-cells *in vitro*, there are conflicting reports of their ability to block B-cell antibody production. One group found that these cells were unable to suppress antibody production (68) while several groups found the opposite (26, 35, 71). Interestingly, Liu et al., found that cTfr suppressive activity increased in correlation with the sequential shift of cTfr from FRIII Foxp3^int^ into a highly-activated Foxp3^hi^ phenotype from healthy donors > active RA patients > patients in remission.

One unifying feature of these studies is that CXCR5^−^ Tregs in circulation are capable of suppressing antibody responses *in vitro* at least as well as, and sometimes better than, cTfr (26, 68). This might be considered an indication that these cells may not be bona fide Tfr. However, whether the ability to suppress humoral responses *in vitro* should be considered a defining property of Tfr is questionable. CXCR5^−^ Treg from the tissues of both humans and mice are fully capable of suppressing B-cell antibody production *in vitro* (12, 26, 34). While certain suppressive mechanisms may skew toward Tfr or eTregs (Figure 3), and may have different roles at different points of the humoral response, it seems likely that none of them are entirely exclusive. This is emphasized by the finding that CTLA-4, IL-10, and IL1-R2 all have roles in both Tfr and Treg suppressive function. This current lack of evidence for a suppressive mechanism which is unique to Tfr, and which might explain any specific ability to suppress humoral immunity by Tfr, suggests that the capacity to suppress B-cells *in vitro* may not be a defining characteristic of Tfr. Instead we favor a model in which the key *in vivo* difference between Tregs and Tfr is not their mechanism of suppression, but rather their localization. Simply, we suggest that both CD25^−^ and CD25^+^ Tfr are able to act at different points of the humoral response from Tregs, because they are in the right place to do so, a distinction that is lost during an *in vitro* assay.

## Tfr in human disease

Due to their relatively recent discovery, the role of Tfr in human disease is not well understood at this time. The proportion of cTfr in human blood may be a direct indicator of the extent of ongoing antibody responses. The total number of cTfr in blood increases after vaccination, while the proportion of them that are CD45RA^+^ drops (68, 69). cTfr are also increased as a proportion of Tregs in patients with ongoing Sjögren's syndrome (SS), and the Tfr/Tfh ratio strongly correlates with both autoantibody production and activated (PD-1^+^ICOS^+^) T-cell infiltration into the minor salivary glands of patients (68, 75). In the context of infection, cTfr expand during chronic viral and parasitic infections such as human immunodeficiency virus (HIV), hepatitis B virus (HBV), hepatitis C virus (HCV), and Schistosoma japonica (76–78) The increase of cTfr cell frequency in patients chronically infected with either HBV or HCV showed strong correlation with serum viral load in both infections. In rheumatoid arthritis, increased percentages of cTfr, decreased percentages of Tfh and a corresponding drop in the ratio of Tfh/Tfr was associated with stable disease and reduced levels of autoantibodies, while active disease was correlated to increased cTfr but no change in the Tfh/Tfr ratio (71). However, treatment may cause cTfr numbers to drop resulting in a high Tfh/Tfr ratio but no clear relationship between cTfr numbers and autoantibodies (79). Similarly to untreated RA patients, the Tfh/Tfr ratio was correlated with autoantibody production in SLE patients, although in this case this was due to a loss of Tfr while the proportion of Tfh remained stable (80). cTfr cell frequency was also reduced in the blood of multiple sclerosis patients and these cells were found to be less suppressive compared to those of healthy controls (69). Together these results suggest that an increased proportion of cTfr in the blood is a marker of ongoing humoral activity and that the Tfh/Tfr ratio may give an indication of autoantibody production. However in the cases of SLE and MS this correlation seems less clear. Whether, as seems possible, this is an indication that autoantibody production in these settings is due to a proliferative defect in cTfr is unclear at this time.

The frequency of cTfr cells in primary immunodeficiency disorders also displays variability. Cunill et al. reported that the smB^−^ (switched memory phenotype B-cell deficient) subset of common variable immunodeficiency (CVID) patients showed remarkable reduction in their blood CXCR5^+^CD25^hi^CD127^low^ Tfr cell numbers (81). Store-operated Ca^2+^ entry (SOCE) via Ca^2+^ release-activated Ca^2+^ (CRAC) channels mediated by STIM and ORAI proteins is an essential signaling pathway in T cells, and it controls both Tfh and Tfr cell differentiation (82). Vaeth et al. demonstrated that frequency of CD45RO^+^Foxp3^+^Helios^+^Tfr-like effector Treg cells is significantly diminished in patients with severe combined immunodeficiency-like disease, characterized by inherited loss-of-function mutations in STIM1 or ORAI1 genes (82) Meanwhile, Jandl et al. found that the percentages of CD4^+^CXCR5^hi^PD-1^hi^CD127^low^ Foxp3^+^ cTfr cells were elevated in the peripheral blood of IL-21R-deficient patients compared to healthy controls (38). Despite this the total frequency of cTfr seem surprisingly resistant to a range of mutations that affect Tfh formation, such as STAT1 and STAT3, IL21R, IL10R, and ICOS (83).

## Tfr in tumors

Increased numbers of activated ICOS^+^CXCR5^+^ Tfr are seen in the blood of non-small cell lung cancer patients although this did not correlated with disease stage (84). Tfr are also enriched in the lymph nodes of diffuse large B cell lymphoma (DLBCL) patients and the proportion of Tfr was reduced in patients with more advanced stage disease (85). It is unclear what the role of Tfr may be in the tumor environment but Tfh like cell infiltration has been demonstrated to be predictive of survival in breast cancer and may drive ectopic germinal centers (86). Although in this case these were CXCR5^−^PD-1^hi^CXCL13 T-helper identified in inflamed joints and breast cancer (described as TfhX13) (87). TfhX13 and Tfh appear closely related, sharing most of the transcriptional programming related to B-cell help but with differing homing capabilities as Tph primarily infiltrate inflamed tissue in a CXCR5 independent manner. It is unclear if Tregs have a direct equivalent for these cells but in breast cancer PD-1^int^ICOS^hi^CXCR5^−^ Tregs are seen infiltrating the same areas suggesting that these may be non-Tfr effector Tregs (87).

## Conclusion

Due to their recent discovery the Tfr field is still young and many important questions about the formation and function of Tfr and their role in a range of antibody driven autoimmune diseases remain unanswered. Recent work examining specific knockout of Tfr suggests that Tfr may be biased to the control of autoantibody responses while having a more subtle role on the production of non-self-antibodies. It seems clear that in mice Tfr readily lose expression of CD25 and this correlates with a germinal center localization a CXCR5^hi^BCL6^hi^PD-1^hi^ phenotype. In humans these cells appear to be less common suggesting that CD25^+^Tfr may have a more dominant role in this setting. However the relative contributions and exact suppressive mechanisms used by CD25^−^Tfr, CD25^+^Tfr and Tregs at different points in the regulation of the humoral immune response remain unclear and may prove hard to separate due to their highly interrelated nature. New tools may be needed to separate these populations, particularly in humans in which our understanding of Tfr biology is limited, making further human Tfr studies an ongoing priority.

## Author contributions

JW conceived and wrote the manuscript and prepared figures. MT and SS contributed to the writing and revision of the manuscript.

### Conflict of interest statement

The authors declare that the research was conducted in the absence of any commercial or financial relationships that could be construed as a potential conflict of interest.
